# Supplementary material for: Global hypo-methylation in a proportion of glioblastoma enriched for an astrocytic signature is associated with increased invasion and altered immune landscape
Source: eLife. 2022 Nov 22;11:e77335. doi: 10.7554/eLife.77335 (PMC9681209; doi:10.7554/eLife.77335)
Supplement: Figure 2—source data 1. [file elife-77335-fig2-data1.zip › Figure_2_source_data_1/Figure_2B/homerResults/motif18.similar.html]

motif18

## Information for motif18

G
C
A
T
A
C
G
T
T
A
G
C
G
A
T
C
G
A
C
T
G
A
C
T
A
C
G
T
C
T
A
G
A
T
G
C
G
C
A
T
  
Reverse Opposite:  

C
G
T
A
T
A
C
G
G
A
T
C
T
G
C
A
C
T
G
A
C
T
G
A
C
T
A
G
A
T
C
G
T
G
C
A
C
G
T
A
  

|  |  |
| --- | --- |
| p-value: | 1e-59 |
| log p-value: | -1.359e+02 |
| Information Content per bp: | 1.786 |
| Number of Target Sequences with motif | 107.0 |
| Percentage of Target Sequences with motif | 4.63% |
| Number of Background Sequences with motif | 2.9 |
| Percentage of Background Sequences with motif | 0.81% |
| Average Position of motif in Targets | 96.6 +/- 59.2bp |
| Average Position of motif in Background | 126.7 +/- 9.7bp |
| Strand Bias (log2 ratio + to - strand density) | 0.1 |
| Multiplicity (# of sites on avg that occur together) | 1.02 |
| Motif File: | file (matrix) reverse opposite |

### Similar de novo motifs found

|  |  |  |  |  |  |  |  |
| --- | --- | --- | --- | --- | --- | --- | --- |
| Rank | Match Score | Redundant Motif | P-value | log P-value | % of Targets | % of Background | Motif file |
| 1 | 0.647 | T A G C G A T C G C T A A T C G G C A T A C G T A G C T A T G C G T A C G C A T A G T C G C A T A C G T A G T C G C A T | 1e-44 | -101.448488 | 2.94% | 0.21% | motif file (matrix) |
| 2 | 0.633 | G A T C G T C A C T G A C T G A C T A G T C A G T C G A C G T A T C G A C G T A T C G A G T C A T C G A C G A T C G T A | 1e-22 | -51.752223 | 4.46% | 1.44% | motif file (matrix) |
| 3 | 0.656 | A C T G A G T C A C G T A C T G C G T A A C T G A C T G C G T A C G T A A C T G | 1e-17 | -39.295790 | 2.94% | 1.06% | motif file (matrix) |
